# Silica nanoparticles inhibit the cation channel TRPV4 in airway epithelial cells

**DOI:** 10.1186/s12989-017-0224-2

**Published:** 2017-11-03

**Authors:** Alicia Sanchez, Julio L. Alvarez, Kateryna Demydenko, Carole Jung, Yeranddy A. Alpizar, Julio Alvarez-Collazo, Stevan M. Cokic, Miguel A. Valverde, Peter H. Hoet, Karel Talavera

**Affiliations:** 1 Department of Cellular and Molecular Medicine, Laboratory of Ion Channel Research, KU Leuven; VIB Center for Brain & Disease Research, Leuven, Belgium; 2 Department of Experimental and Health Sciences, Laboratory of Molecular Physiology and Channelopathies, Universitat Pompeu Fabra, Barcelona, Spain; 3 KU Leuven BIOMAT, Department of Oral Health Sciences, KU Leuven & Dentistry University Hospitals Leuven, Leuven, Belgium; 4 Department of Public Health and Primary Care, KU Leuven, Leuven, Belgium; 5 Present address: Department of Cardiovascular Sciences, Laboratory of Experimental Cardiology, Leuven, KU Belgium

**Keywords:** silica nanoparticles, TRPV4, GSK1016790A, epithelial cells, ciliary beat frequency

## Abstract

**Background:**

Silica nanoparticles (SiNPs) have numerous beneficial properties and are extensively used in cosmetics and food industries as anti-caking, densifying and hydrophobic agents. However, the increasing exposure levels experienced by the general population and the ability of SiNPs to penetrate cells and tissues have raised concerns about possible toxic effects of this material. Although SiNPs are known to affect the function of the airway epithelium, the molecular targets of these particles remain largely unknown. Given that SiNPs interact with the plasma membrane of epithelial cells we hypothesized that they may affect the function of Transient Receptor Potential Vanilloid 4 (TRPV4), a cation-permeable channel that regulates epithelial barrier function. The main aims of this study were to evaluate the effects of SiNPs on the activation of TRPV4 and to determine whether these alter the positive modulatory action of this channel on the ciliary beat frequency in airway epithelial cells.

**Results:**

Using fluorometric measurements of intracellular Ca^2+^ concentration ([Ca^2+^]_i_) we found that SiNPs inhibit activation of TRPV4 by the synthetic agonist GSK1016790A in cultured human airway epithelial cells 16HBE and in primary cultured mouse tracheobronchial epithelial cells. Inhibition of TRPV4 by SiNPs was confirmed in intracellular Ca^2+^ imaging and whole-cell patch-clamp experiments performed in HEK293T cells over-expressing this channel. In addition to these effects, SiNPs were found to induce a significant increase in basal [Ca^2+^]_i_, but in a TRPV4-independent manner. SiNPs enhanced the activation of the capsaicin receptor TRPV1, demonstrating that these particles have a specific inhibitory action on TRPV4 activation. Finally, we found that SiNPs abrogate the increase in ciliary beat frequency induced by TRPV4 activation in mouse airway epithelial cells.

**Conclusions:**

Our results show that SiNPs inhibit TRPV4 activation, and that this effect may impair the positive modulatory action of the stimulation of this channel on the ciliary function in airway epithelial cells. These findings unveil the cation channel TRPV4 as a primary molecular target of SiNPs.

## Background

Synthetic amorphous SiNPs are extensively used due to its interesting physico-chemical properties, low cost and relatively easy production. This material has many applications in industrial manufacturing, cosmetics, biotechnology, medicine, and food, pharmaceutical and chemical industries [1–4]. SiNPs are widely used in consumer products and as a consequence, human exposure to this nanomaterial has highly increased. However, there is very little information available about the risks associated to the exposure to this nanomaterial [5].

It is known that SiNPs can penetrate cells, interacting with the plasma membrane, intracellular structures and organelles, thereby posing potential health threats [6–8]. The toxicity generated by nanoparticles has been related to an increased generation of reactive oxygen species (ROS) [9, 10]. This results in oxidative stress, mitochondrial perturbation and the generation of inflammatory mediators leading to cell dysfunction and apoptosis [2, 3, 11–19].

One of the main entry pathways of nanoparticles into the body is the epithelium of the airways. In addition to its function in gas exchange, the respiratory epithelium protects the body against hazardous environmental substances and pathogens, constituting an active diffusion barrier, and supporting the mechanisms of mucociliary clearance and recruitment of inflammatory cells [20–22]. Several cellular responses to SiNPs in the airways have been reported. Rabiolli et al. demonstrated that SiNPs induce lung inflammation through the stimulation of IL-1β production by alveolar macrophages [23]. Skuland et al. showed evidence of pro-inflammatory responses induced by amorphous SiNPs in lung epithelial cells [24]. Delaval et al. reported that SiNPs pre-exposure in pneumonia induced by *Pseudomonas aeruginosa* increases lung permeability and enhance mortality [25], and Kasper et al. showed inflammatory and cytotoxic responses such as DNA damage, hypoxia and ER-stress induced by SiNPs in an alveolar-capillary co-culture model [18].

However, little is known about the influence of SiNPs on specific molecular targets and cell signaling events, especially at the level of the plasma membrane. In this study we hypothesized that SiNPs may affect the function of TRPV4, a Ca^2+^-permeable cation channel expressed in airway epithelial cells. This channel plays a role in the transduction of physical and chemical stimuli into Ca^2+^ signals that regulate ciliary beat frequency and mucociliary transport [26, 27]. Moreover, TRPV4 contributes to the barrier integrity in the lung and to the regulation of endothelial and epithelial permeability [28, 29], and has been implicated in the modulation of the respiratory function and proposed as target for the treatment of respiratory diseases such chronic obstructive pulmonary disease and asthma [30–33].

We used intracellular Ca^2+^ imaging and patch-clamp to evaluate the effects of SiNPs on TRPV4 activation. We found that SiNPs inhibit the activation of native TRPV4 channels in human and mouse airway epithelial cells, as well as recombinant TRPV4 in the heterologous expression system HEK293T. Furthermore, SiNPs inhibited the TRPV4-mediated increase in ciliary beat frequency in mouse airway epithelial cells. TRPV4 emerges therefore as a defined molecular target of SiNPs, with possible deleterious consequences for epithelial barrier function.

## Methods

### Ludox® SiNPs

SM30 Ludox® SiNPs were purchased from Sigma-Aldrich (Bornem, Belgium) as the commercial source of 30% wt suspension in H_2_O. For the biological experiments the nanoparticle suspension was diluted to the desired concentrations in Krebs solution containing (in mM): 150 NaCl, 6 KCl, 1 MgCl_2_, 1.5 CaCl_2_, 10 glucose, 10 4-(2-hydroxyethyl)-1-piperazineethanesulfonic acid (HEPES) and titrated to pH 7.4 with NaOH.

### Dynamic Light Scattering (DLS) and Zeta potential

The stock suspension of SiNPs particles was diluted in water to 30 μg/ml. DLS and Zeta potential measurements were performed with a Brookhaven 90 Plus/ZetaPlus instrument (Brookhaven Instruments Ltd, Redditch, UK). DLS measurements were performed using a NanoParticle Size Distribution Analyser (scattering angle 90 u, wavelength 659 nm, power 15 mW). Correlation functions were analyzed using the Clementine package (maximum entropy method) for Igor Pro 6.02A (WaveMetrics, Portland, OR, USA).

Zeta potential measurements were done by applying electrophoretic light scattering. A primary and reference beam (659 nm, 35 mW), modulated optics and a dip-in electrode system were used. The frequency shift of scattered light (relative to the reference beam) from a charged particle moving in an electric field is related to the electrophoretic mobility of the particle. The Smoluchowski limit was used to calculate the Zeta potential from the electrophoretic mobility.

### Transmission Electron Microscopy (TEM)

Suspensions (5 μl of stock suspension and 30 μg/ml) of the SiNPs particles were applied on formvar-coated cupper mesh grids (drop on grid). After drying overnight (25 °C in the dark), the particles were characterized by TEM (JEOL JEM-1200 EX-II, Tokyo, Japan).

### Endosafe-PTS

We used the Endosafe-PTS LAL assay for FDA-licensed endotoxin detection. The cartridges contained four channels to which LAL reagent and a chromogenic substrate were applied. Two of these channels contained also an endotoxin spike that served as positive control. The sensitivity of the assay was 0.05 EU/ml.

### Cell culture

Human bronchial epithelial cell line, 16HBE, were grown in Dulbecco's modified Eagle's medium: nutrient mixture F-12 (DMEM/F-12) containing 5% (v/v) fetal calf serum (FCS), 2 mM L-glutamine, 2 U/ml penicillin and 2 mg/ml streptomycin at 37 °C in a humidity-controlled incubator with 5% CO_2_ and were seeded on 18-mm glass cover slips coated with poly-L-lysine (0.1 mg/ml).

Human embryonic kidney cells, HEK293T, were grown in Dulbecco's modified Eagle's medium (DMEM) containing 10% (v/v) fetal calf serum (FCS), 2 mM L-glutamine, 2 U/ml penicillin, 1% non-essential amino acids (Invitrogen, Erembodegem - Aalst, Belgium) and 2 mg/ml streptomycin at 37 °C in a humidity-controlled incubator with 10% CO_2_ and were seeded on 18-mm glass cover slips coated with poly-L-lysine (0.1 mg/ml). For intracellular Ca^2+^ imaging and patch-clamp experiments, HEK293T cells were transiently transfected with mouse TRPV4 in the CAGGSM2/Ires/GFP/R1R2 vector, using Mirus TransIT-293 (Mirus Corporation; Madison, WI, USA). In all experiments, transfected cells were identified by green fluorescent protein (GFP) expression.

### Animals

C57Bl/6J male mice from 8-12 weeks old were used for the experiments. The animals were maintained under standard conditions with a maximum of four animals per cage on a 12-h light/12-h dark cycle and with food and water ad libitum.

### Culture of mouse tracheal epithelial cells

Mouse tracheal epithelial cells (mTEC) were isolated following the protocol described by Lam et al. [32], and seeded on 18-mm glass cover slips coated with collagen solution containing 50 μg/ml collagen (type I solution from rat tail, Sigma-Aldrich). Cells were grown for 2-3 days in the appropriate proliferation medium and maintained at 37 °C in a humidity-controlled incubator with 5% CO_2_.

### Intracellular Ca^2+^ imaging experiments

Ca^2+^-imaging experiments were conducted with the ratiometric fluorescent indicator Fura-2 acetoxymethyl (AM) ester. Cells were incubated with 2 μM Fura-2 AM for 30 min at 37 °C. Bath solutions were perfused by gravity via a multi-barreled pipette tip with a single outlet of 0.8 mm inner diameter. This system allows full exchange of the medium bathing the recorded cell in less than 2-4 s. For recording in control condition cells were rinsed with Krebs solution. The [Ca^2+^]_i_ was monitored through the ratio of fluorescence measured upon alternating illumination at 340 and 380 nm using an MT-10 illumination system and the xcellence pro software (Olympus, Planegg, Germany). All experiments conducted in the native 16HBE and mTEC cells were performed at 35 °C. Experiments in HEK293T cells were performed at 25 °C because at 35 °C the TRPV4-transfected cells were heavily overloaded with Ca^2+^ in basal condition.

The concentration-dependent effects of SiNPs on basal [Ca^2+^]_i_ of 16HBE cells was fit with a Hill function of the form:

$$ \varDelta \left[{Ca}^{2+}\right]=\varDelta {\left[{Ca}^{2+}\right]}_{Max}\frac{{\left[ SiNPs\right]}^H}{{\left[ SiNPs\right]}^H+{EC}_{50}^H} $$

where ∆[Ca^2+^]_*Max*_ is the maximal amplitude of the response to SiNPs, [SiNPs] is the concentration of SiNPs, *EC*_*50*_ is the effective concentration and *H* is the Hill coefficient.

The concentration-dependent effects of SiNPs on the Ca^2+^ responses to the TRPV4 agonist GSK1016790A were fit with a Hill function of the form:

$$ \varDelta \left[{Ca}^{2+}\right]=\left(\varDelta {\left[{Ca}^{2+}\right]}_{Max}-\varDelta {\left[{Ca}^{2+}\right]}_{Inf}\right)\frac{{\left[ SiNPs\right]}^H}{{\left[ SiNPs\right]}^H+{IC}_{50}^H}+\varDelta {\left[{Ca}^{2+}\right]}_{Inf} $$

where ∆[Ca^2+^]_*Max*_ is the amplitude of the response in the absence of SiNPs, ∆[Ca^2+^]_*Inf*_ is the amplitude of the response the presence of saturating concentrations of SiNPs, [SiNPs] is the concentration of SiNPs, *IC*_*50*_ is the effective inhibitory concentration and *H* is the Hill coefficient.

### Patch-clamp experiments

Whole-cell voltage-clamp recordings were performed at 35 °C with standard patch pipettes (2-3 MΩ resistance) pulled using a DMZ-Universal puller (Zeitz Instruments, Augsburg, Germany). The pipette solution contained (in mM): 2 ATPNa_2_, 5 EGTA, 10 HEPES, 1 MgCl_2_, 135 CsCl_2_ (292 mOsm/kg; pH 7.2, adjusted with CsOH). For perforated patch experiments, 250 μg/ml of Amphotericin B was added to the pipette solution and data were collected after the access resistance reached stable values of ~15 MΩ.

An Ag-AgCl wire was used as reference electrode. The cover slips with cells were placed in the stage of an inverted microscope (Olympus IX70, Tokyo, Japan) and stabilized for a few minutes in Krebs solution, containing (in mM): 150 NaCl, 6 KCl, 1 MgCl_2_, 1.5 CaCl_2_, 10 glucose, and 10 HEPES and titrated to pH 7.4 with NaOH. The control bath solution was kept at room temperature and contained (in mM): 140 NaCl, 1.3 MgCl_2_, 2.4 CaCl_2_, 10 HEPES, 10 glucose and (311 mOsm/kg; pH 7.4, adjusted with NaOH). Bath solutions were perfused by gravity via a multi-barreled pipette. A bath solution in which all cations were isotonically substituted by NMDG^+^ (N-methyl-D-glucamine) was used to monitor the size of the leak currents during the patch-clamp recordings [34]. Current signals were recorded using the patch-clamp technique by using an EPC-7 (LIST Electronics, Darmstadt, Germany) amplifier and the Clampex 9.0 software program (Axon instruments, Sunnyvale, CA, USA). Currents were acquired at 10 kHz, filtered at 2 kHz, and stored for off-line analysis on a personal computer. In order to minimize voltage errors, the series resistance was compensated by 30-50% and the capacitance artifact was reduced using the amplifier circuitry. Membrane TRPV4 currents were elicited by a 600 ms long voltage ramp from -100 mV to +100 mV every 5 s with a holding potential of 0 mV.

Patch-clamp data was analyzed with the WinASCD software written by Dr. Guy Droogmans and Origin 7.0 (OriginLab Corporation, Northampton, MA, USA). The concentration dependence of TRPV4 current amplitude was fit with a Hill function of the form:

$$ \varDelta I=\left(\varDelta {I}_{Max}-\varDelta {I}_{Inf}\right)\frac{{\left[ SiNPs\right]}^H}{{\left[ SiNPs\right]}^H+{IC}_{50}^H}+\varDelta {I}_{Inf} $$

where ∆*I*_*Max*_ is the current density increase in the absence of SiNPs, *∆I*_*Inf*_ is the current density increase in the presence of saturating concentrations of SiNPs, [SiNPs] is the concentration of SiNPs, *IC*_*50*_ is the effective inhibitory concentration and *H* is the Hill coefficient.

### Ciliary beat frequency (CBF) measurements

CBF was measured in primary cultures ciliated cells using with a high-speed digital imaging system as previously described [26]. Briefly, phase-contrast images (512 × 512 pixels) were collected at 120–135 frames per second with a high speed CCD camera using a frame grabber (Infaimon, Barcelona, Spain) and recording software from Video Savant (IO Industries, London, ON, Canada). The ciliary beat frequency was determined from the frequency of variation in light intensity of the image as a result of repetitive motion of cilia.

### Reagents

All chemicals were purchased from Sigma-Aldrich (Bornem, Belgium).

### Statistics

Data are given as mean ± standard error of the mean. Comparisons tests are indicated in the text were appropriate. Statistical significance were taken at *P* < 0.05 or *P* < 0.01.

## Results

### Characterization of the SM30 Ludox® SiNPs

Analysis of the SiNPs by DLS showed a single population of average size 10.2 nm (P10: 8.1 nm - P90: 11.8). The particles had a Zeta-potential of 20 ± 3 mV. TEM analysis of undiluted samples showed large aggregates, but in the diluted samples only a few aggregates could be found, and the particles appeared as spherical entities. No endotoxin contamination was detected in 30 μg/ml SiNPs dilutions.

### Silica NPs inhibit TRPV4 activation in cultured human airway epithelial cells

**Fig. 1:**
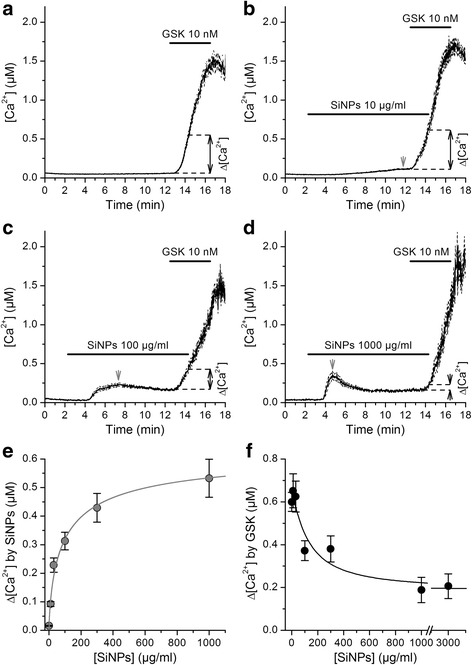
SiNPs inhibit TRPV4 activation in 16HBE cells. **a**, **b**, **c**, **d** Effects of the TRPV4 agonist GSK1016790A on the [Ca^2+^]_i_ in the absence (**a**, *n* = 333) or in the presence of SiNPs 10 μg/ml (**b**, *n* = 234), 100 μg/ml (**c**, *n* = 163) and 1000 μg/ml (**d**, *n* = 114). The thick continuous traces correspond to the average responses and the thin dashed traces correspond to the mean plus/minus the standard errors. The dashed lines and black arrows indicate the amplitude of the average Ca^2+^ responses recorded after 2 min application of GSK1016790A, with respect to the immediate previous basal [Ca^2+^]_i_. The gray arrows point to the peak of basal Ca^2+^ responses to SiNPs. **e** Concentration dependence of the maximal amplitude of basal Ca^2+^ responses induced by the application of SiNPs. **f** Average amplitude of responses to 10 nM GSK1016790A when applied in the presence of SiNPs at different concentrations. In (**e**) and (**f**) the solid lines represent fits with Hill functions (see Methods)

To determine whether SiNPs modulate native human TRPV4 channels we used fluorometric measurements of [Ca^2+^]_i_ in cultured human bronchial epithelial 16HBE cells, which were reported to express this channel [34–37]. We found that 10 nM GSK1016790A induced intracellular Ca^2+^ responses in 100% (n = 333) of these cells, indicating for a prevalent functional expression of TRPV4 (Fig. 1a). Extracellular application of SiNPs increased the basal [Ca^2+^]_i_in a concentration-dependent manner (Fig. 1b-d), which was characterized by an *EC*_*50*_ of 99 ± 13 μg/ml, a Hill coefficient of 0.71 ± 0.07 and a maximal response of 0.6 ± 0.1 μM (Fig. 1e). To evaluate the effect of SiNPs on TRPV4 activation we compared the amplitude of the [Ca^2+^]_i_ responses measured 2 min after application of 10 nM GSK1016790A, in the absence and in the presence of nanoparticles. SiNPs induced a concentration-dependent inhibition of the responses to GSK1016790A, with an *IC*_*50*_ of 130 ± 40 μg/ml and a Hill coefficient of -1.2 ± 0.4 (Fig. 1f). Of note, SiNPs failed to completely abolish the response to GSK1016790A up to a concentration of 3000 μg/ml, leaving ~30% of the response to the channel agonist.

### SiNPs increase basal [Ca^2+^]_i_ in a TRPV4-independent manner

**Fig. 2:**
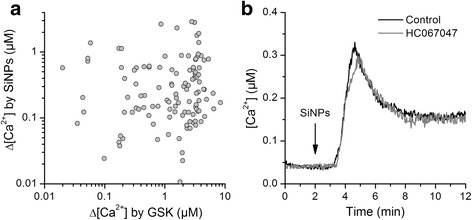
TRPV4 does not mediate the increase in basal [Ca^2+^]_i_ induced by SiNPs in 16HBE cells. **a** Lack of correlation of the amplitudes of the intracellular Ca^2+^ responses to 300 μg/ml SiNPs and to the TRPV4 agonist GSK047067 (10 nM). **b** Effect of 300 μg/ml SiNPs in the absence (Control) and in the presence of the TRPV4 inhibitor HC067047 (10 μM). The lines represent average responses of 120 and 164 cells in control and HC067047, respectively

It has been previously suggested that TRPV4 is implicated in intracellular Ca^2+^ responses to SiNPs in a cell subpopulation of the GT1-7 neuron-derived cell line [38]. Thus, we tested whether TRPV4 is involved in the [Ca^2+^]_i_ increases triggered by SiNPs in 16HBE cells. First, we determined whether the amplitude of the responses to SiNPs correlated with the amplitude of the responses to GSK1016790A, i.e., with the level of functional expression of TRPV4 in each cell. We found that this was not the case, with correlation values (R) of 0.074 (Fig. 2a). This value is lower than those we have previously found for the correlation between the amplitudes of responses to very low and high concentrations of GSK1016790A [39]. The average increase in basal [Ca^2+^]_i_ elicited by 300 μg/ml SiNPs in 16HBE was not significantly different in the absence (0.40 ± 0.04 μM) and in the presence of the specific TRPV4 blocker HC067047 [40] (0.43 ± 0.05 μM; P = 0.64; Fig. 2b). These data demonstrate that TRPV4 does not mediate the basal Ca^2+^ responses triggered by SiNPs.

### An increase in basal [Ca^2+^]_i_ does not inhibit a subsequent TRPV4 activation

**Fig. 3:**
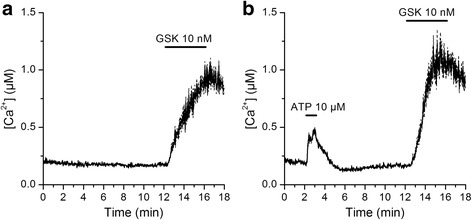
Stimulation of 16HBE cells with ATP potentiates a subsequent activation of TRPV4. **a**, **b** Effects of the TRPV4 agonist GSK1016790A on the [Ca^2+^]_i_ of HEK293T cells transfected with mouse TRPV4, in control (**a**, *n* = 253) or after extracellular perfusion of ATP (**b**, *n* = 266). The thick continuous traces correspond to the average responses and the thin dashed traces correspond to the mean plus/minus the standard errors

Next, we determined if an increase in basal [Ca^2+^]_i_ such as that induced by SiNPs could cause a decrease in TRPV4 activation. For this we tested the effect of extracellular application of ATP on a subsequent response of 16HBE cells to 10 nM GSK1016790A. We found that ATP triggered a robust intracellular Ca^2+^ signal, and that this did not reduced, but rather increased the amplitude of the TRPV4 response measured at 2 min of GSK1016790A application (0.54 ± 0.05 vs. 0.82 ± 0.08 in control and after ATP application, respectively, *P* = 0.005; Fig. 3a, b).

### SiNPs inhibit TRPV4 activation in mouse tracheal epithelial (mTEC) cells

**Fig. 4:**
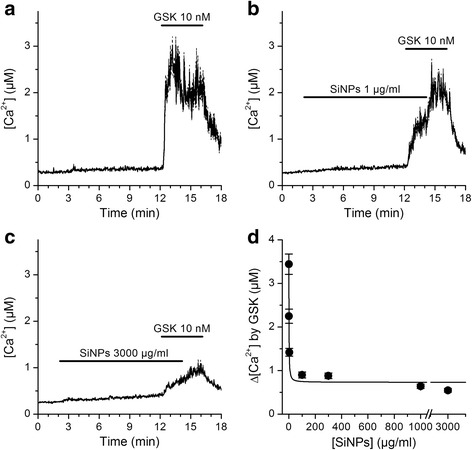
SiNPs inhibit TRPV4 activation in mTEC. **a**, **b**, **c**, **d** Effects of the TRPV4 agonist GSK1016790A on the [Ca^2+^]_i_ in the absence (**a**, *n* = 127) or in the presence of SiNPs 1 μg/ml (**b**, *n* = 243) and 3000 μg/ml (**c**, *n* = 349). The thick continuous traces correspond to the average responses and the thin dashed traces correspond to the mean plus/minus the standard errors. **d** Average amplitude of responses to 10 nM GSK1016790A when applied in the presence of SiNPs at different concentrations. The solid line represents a fit with a Hill function (see Methods)

In order to determine whether the effect of SiNPs is conserved for native mouse TRPV4 we used primary cultured mouse tracheal epithelial cells. Application of 10 nM GSK1016790A triggered intracellular Ca^2+^ signals in 94.4% (187 out of 198) of these cells (Fig. 4a), consistent with a previous report on the functional expression of TRPV4 channels in these cells [26]. SiNPs induced a concentration-dependent inhibition of the responses to GSK1016790A, with an *IC*_*50*_ of 1.2 ± 0.2 μg/ml and a Hill coefficient of -1.1 ± 0.3 (Fig. 4b-d). As observed in 16HBE cells, application of SiNPs at high concentrations did not abolish the response to the TRPV4 agonist, but left ~20% of the maximal response.

### SiNPs inhibit activation of recombinant TRPV4

**Fig. 5:**
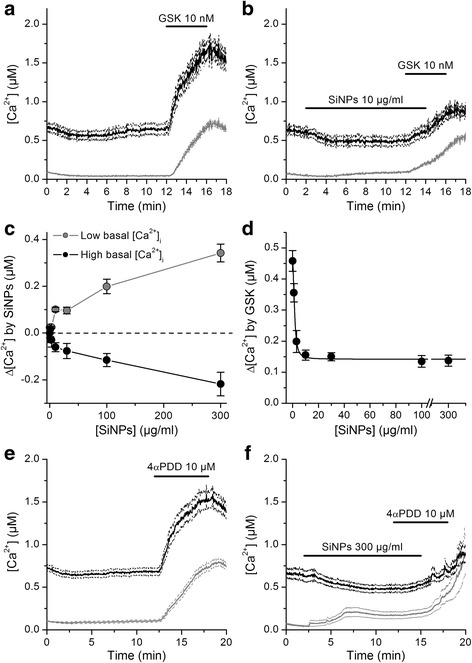
SiNPs inhibit activation of mouse TRPV4 heterologously expressed in HEK293T cells. **a**, **b** Effects of the TRPV4 agonist GSK1016790A on the [Ca^2+^]_i_ in the absence (**a**, *n* = 127) or in the presence of 10 μg/ml SiNPs (**b**, *n* = 243). The thick continuous traces correspond to the average responses and the thin dashed traces correspond to the mean plus/minus the standard errors. The black and grey traces correspond to cells with high or low basal [Ca^2+^]_i_, respectively (see text). **c** Concentration-dependent effects on SiNPs on the intracellular Ca^2+^ levels in cells with low and high basal Ca^2+^. **d** Average amplitude of responses to 10 nM GSK1016790A when applied in the presence of SiNPs at different concentrations. The solid line represents a fit with a Hill function (see Methods). **e**, **f** Effects of the TRPV4 agonist 4αPDD on the [Ca^2+^]_i_ in the absence (**a**, *n* = 347) or in the presence of SiNPs (**b**, *n* = 164). The traces are color-coded as in panels (**a**) and (**b**)

To test whether SiNPs inhibit activation of TRPV4 in a heterologous expression system we performed intracellular Ca^2+^ imaging experiments in HEK293T cells transiently transfected with the mouse channel isoform. These cells showed a wide spectrum of basal [Ca^2+^]_i_, a fact that we ascribe to the variable efficacy of TRPV4 transfection in each cell and the constitutive activity of this Ca^2+^-permeable channel. Analysis of the distribution of these values suggested the presence of two cell populations, which could be divided using a cutoff value of 250 nM. Both groups of cells responded robustly to 10 nM GSK1016790A (Fig. 5a).

Application of SiNPs induced a concentration-dependent increase of [Ca^2+^]_i_ in cells with low basal [Ca^2+^]_i_ (Fig. 5b, c), an effect reminiscent of that we observed in 16HBE cells (Fig. 1b-e). In contrast, SiNPs reduced [Ca^2+^]_i_ in cells with high basal Ca^2+^ levels (Fig. 5b, c), which may be an indicative of an inhibitory effect of the SiNPs on the basal activity of TRPV4. In both groups SiNPs reduced the response to GSK1016790A with an *IC*_*50*_ of 1.44 ± 0.06 μg/ml and a Hill coefficient of -2.0 ± 0.16 (Fig. 5d). Again, we found that application of SiNPs at high concentrations left ~30% of the response to the TRPV4 agonist.

Next, we determined whether SiNPs inhibit the activation of TRPV4 by another synthetic chemical agonist, 4α-phorbol 12,13-didecanoate (4αPDD). We found that SiNPs (300 μg/ml) strongly inhibited the response to 4αPDD (Fig. 5e, f), showing that their effect is not exclusive for channel activation with GSK1016790A.

**Fig. 6:**
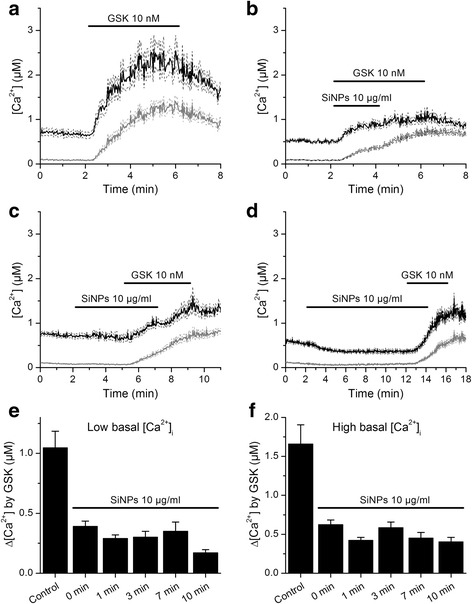
The pre-application time of SiNP has no significant effect on the magnitude of inhibition of TRPV4 response to GSK1016790A. **a** Effects of the TRPV4 agonist GSK1016790A on the [Ca^2+^]_i_ of HEK293T cells transfected with mouse TRPV4. **b**, **c**, **d** TRPV4 responses to GSK1016790A after pre-application of SiNPs during different periods (0, 3 and 10 min for panels **b**, **c** and **d**, respectively). The thick continuous traces correspond to the average responses and the thin dashed traces correspond to the mean plus/minus the standard errors. The black and grey traces correspond to cells with high or low basal [Ca^2+^]_i_, respectively (see text). **e**, **f** Average amplitude of responses to 2 min applications of GSK1016790A in control and after pre-application of SiNPs for different periods (*n* = 105 - 385)

In all experiments described above we allowed sufficient time for the SiNPs effects on basal [Ca^2+^]_i_ to roughly reach a steady-state (~10 min). However, we were also interested in estimating the time required for these particles to reduce TRPV4 activation. Thus, we performed a series of experiments in which we varied the time of application of SiNPs before stimulating TRPV4 with 10 nM GSK1016790A. This time varied from zero (simultaneous application of SiNPs and GSK1016790A) to 10 min. We found that TRPV4 responses were significantly smaller in the presence of SiNPs (*P* < 0.05) and that the strength of inhibition was not significantly different when comparing across the various pre-application times tested (Tukey's Multiple Comparison Test; Fig. 6). This indicates that the inhibitory action of these particles on TRPV4 activation was prior to the full activation of TRPV4 by GSK1016790A (~2 - 3 min).

**Fig. 7:**
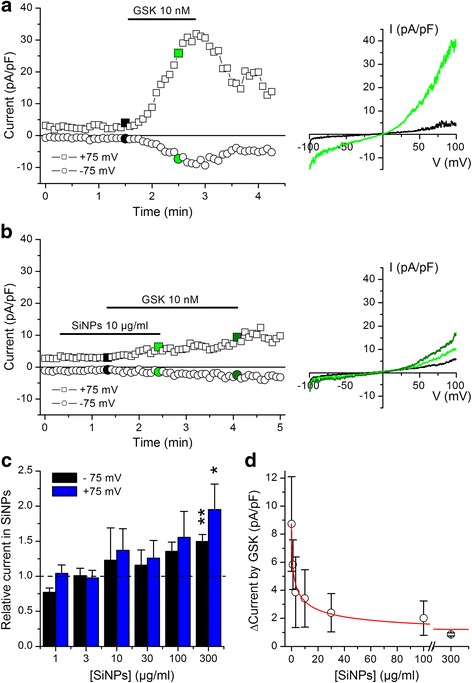
SiNPs inhibit activation of TRPV4 currents in whole-cell patch-clamp recordings. **a**, **b**) Experiments in TRPV4-transfected HEK293T cells showing the effects of the TRPV4 agonist GSK1016790A in the absence (**a**) and in the presence (**b**) of SiNPs. The data points represent the amplitude of the currents measured at -75 and +75 mV. The colored data points correspond to the current traces displayed on the panels shown on the right. **c** Average amplitude of currents recorded at -75 and +75 mV during application of SiNPs. For every cell these values were normalized to the amplitude measured in control condition (*n* = 3 - 10; * and ** indicate *P* < 0.05 and *P* < 0.01, respectively; t test for comparison to 1). **d** Concentration-dependent inhibitory effect of SiNPs on the amplitude of TRPV4 currents measured at -75 mV during 1 min application of GSK1016790A. The solid line represents the fit with a Hill function (see Methods)

To directly test the effects of SiNPs on TRPV4 we performed whole-cell patch-clamp experiments (Fig. 7a, b). We recorded currents during the application of repetitive voltage ramps applied from -100 to +100 mV. Application of SiNPs at increasing concentrations had a tendency to augment the amplitude of basal currents, but this was statistically significant only at 300 μg/ml (Fig. 7c). To evaluate the effect of SiNPs on TRPV4 we compared the amplitude of the current responses measured 1 min after application of 10 nM GSK1016790A, in the absence and in the presence of nanoparticles (Fig. 7a and b). The response to GSK1016790A was significantly reduced when this compound was applied in the presence of SiNPs. This effect was dependent on the concentration of SiNPs, and was characterized by an *IC*_*50*_ of 2.4 ± 0.5 μg/ml, a Hill coefficient of -0.54 ± 0.07 and minimum value of 0.7 ± 0.2 pA/pF, for the currents measured at -75 mV (Fig. 7d).

**Fig. 8:**
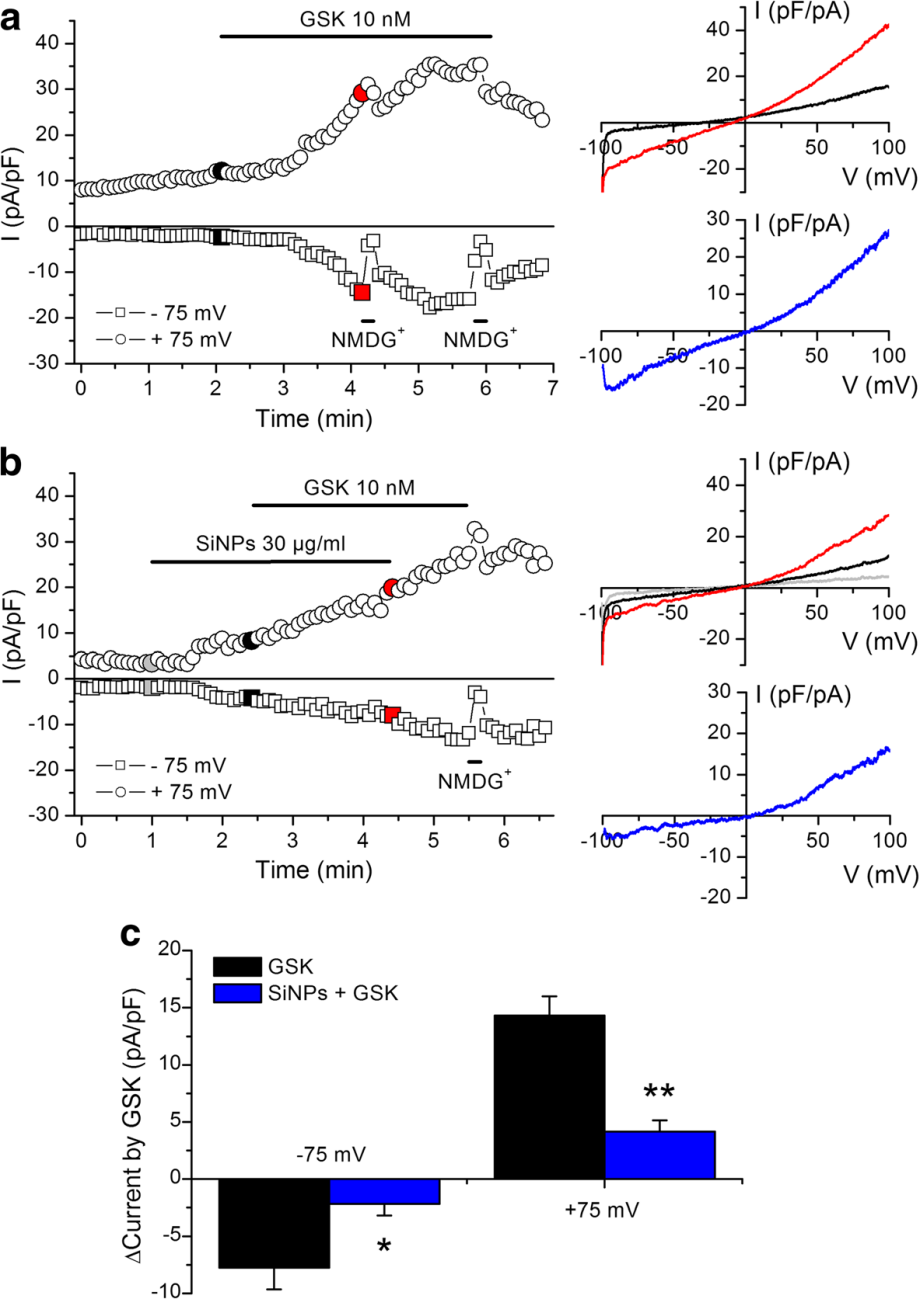
SiNPs inhibit activation of TRPV4 currents in perforated patch-clamp recordings. **a**, **b** Experiments in TRPV4-transfected HEK293T cells showing the effects of the TRPV4 agonist GSK1016790A in the absence (**a**) and in the presence (**b**) of SiNPs. The data points represent the amplitude of the currents measured at -75 and +75 mV. The colored data points correspond to the current traces displayed on the panels shown on the right. **c** Average increase in the amplitude of currents (recorded at -75 and +75 mV) induced by the application of 10 nM GSK1016790A during 2 min, in the absence (*n* = 5) and in the presence (*n* = 6) of 30 μg/ml SiNPs. The * and ** symbols indicate *P* < 0.05 and *P* < 0.01; unpaired t test

To test whether the inhibition of TRPV4 activation is observed also in experimental conditions in which the intracellular milieu is better preserved we performed perforated patch-clamp experiments using Amphotericin B in the patch pipette (Fig. 8a, b). We found that extracellular application of SiNPs (30 μg/ml) significantly reduced the response of TRPV4 current to 10 nM GSK1016790A (Fig. 8c).

### Effects of SiNPs on the capsaicin receptor TRPV1

**Fig. 9:**
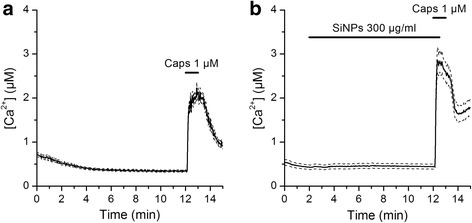
Effects of SiNPs on the capsaicin receptor TRPV1. **a**, **b** Effects of the TRPV1 agonist capsaicin on the [Ca^2+^]_i_ of HEK293T cells transfected with mouse TRPV1, in the absence (**a**, *n* = 200) or in the presence of SiNPs (**b**, *n* = 68). The thick continuous traces correspond to the average responses and the thin dashed traces correspond to the mean plus/minus the standard errors

Next, we performed intracellular Ca^2+^ imaging experiments to determine the effects of 300 μg/ml SiNPs on the response of TRPV1 to its specific agonist capsaicin (1 μM). TRPV1 is the founding member of the vanilloid subfamily of TRP channels, and its amino acid sequence has 42.3% identity and 58.9% similarity with that of TRPV4 (EMBOSS Needle application for Protein Alignment; http://www.ebi.ac.uk/Tools/psa/emboss_needle/help/index-protein.html). We found that the responses of HEK293T cells transfected with mouse TRPV1 to capsaicin were increased by 44% in the presence of SiNPs (P = 0.0011; Fig. 9).

### SiNPs inhibit TRPV4-mediated increase of ciliary beat frequency in airway epithelial cells

**Fig. 10:**
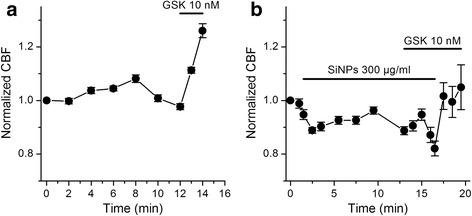
SiNPs inhibit the increase in ciliary beat frequency induced by activation of TRPV4 in mTEC. **a** Increase of CBF induced by application of the TRPV4 agonist GSK1016790A. **b** Lack of effect of GSK1016790A on the CBF in the presence of SiNPs

The cilia of airway epithelial cells are considered to be sensory organelles with the ultimate function of sweeping mucous loaded with pollutants and pathogens out of the airways. TRPV4 is expressed in the cilia, and has been proposed to regulate mucociliary transport by transducing physical and chemical stimuli such as viscosity or fluid tonicity into a Ca^2+^ signal that enhances ciliary beat frequency [26, 27, 41, 42]. Thus, to determine whether the inhibition of TRPV4 by SiNPs has a correlate at the level of a cellular function, we determined the effects of these nanoparticles on the response of cilia to GSK1016790A. Application of 10 nM GSK1016790A in control condition induced a significant 26 ± 3% increase in CBF (*n* = 22; *P* < 10^-4^; paired t test; Fig. 10a). This effect was very similar to that reported by Alenmyr et al. in human nasal epithelial cells [43]. Application of 300 μg/ml SiNPs modestly reduced the basal CBF (~10%; n = 34; *P* < 10^-4^; paired t test; Fig. 10b) and fully abrogated the response to GSK1016790A (*n* = 34; *P* = 0.41; paired t test between CBF immediately before and after 3 min application of the TRPV4 agonist). Washout of SiNPs in the presence of the TRPV4 agonist led to an increase in CBF (*n* = 34; *P* = 0.014; paired t test). Because SiNPs reduced the basal CBF only slightly and fully inhibited the response to GSK1016790A we argue that the latter effect was mainly mediated by inhibition of TRPV4, and not by an unspecific effect of the nanoparticles on other mechanisms regulating the CBF.

## Discussion

Despite the current advances in the characterization of the toxicological properties of SiNPs, little is known about how this material interacts with specific cellular components. Under the plausible assumption that SiNPs interact primarily with the plasma membrane of epithelial cells, in this study we evaluated the effects on TRPV4, a cation-permeable channel that is highly enriched in these cells.

In essence, we found that SiNPs inhibit intracellular Ca^2+^ signals triggered by activation of native TRPV4 channels in human and mouse airway epithelial cells. TRPV4 inhibition by SiNPs was confirmed with Ca^2+^ imaging and direct measurements of TRPV4 currents in the heterologous expression system HEK293T. In sharp contrast to these results, we show that SiNPs enhanced the activation of TRPV1, demonstrating that these particles have a specific inhibitory action on TRPV4 channels. Finally, we found that SiNPs abrogate the increase in ciliary beat frequency induced by TRPV4 activation in mouse airway epithelial cells.

The comparison of the data obtained in mTEC and HEK293T cells transfected with mouse TRPV4 indicate that SiNPs have very similar effects on the responses to GSK1016790A, with *IC*_*50*_ values around 1 μg/ml. In contrast, SiNPs appeared to be much less effective in 16HBE cells, with a 100-fold higher *IC*_*50*_ value. This may indicate that human TRPV4 is less sensitive than the mouse isoform. Nevertheless, the SiNPs concentrations required to inhibit TRPV4 mediated responses in the human-derived cells (100 - 3000 μg/ml) are in the same range or lower than those used in cytotoxicity and cytokine release *in vitro* experiments performed in previous studies (25 - 6000 μg/ml [18, 23, 24]. Moreover, we observed the inhibitory effect on TRPV4 in a matter of minutes, which represents a time scale 3- to 150-fold shorter than that of those previous reports. This strongly suggests TRPV4 as a primary and sensitive target of SiNPs.

As for the mechanism underlying the effects of SiNPs, it may be speculated that these particles somehow disrupt the binding site of GSK1016790A. SiNPs are roughly the same size of the whole channel protein, and more than twice the size of the length of the channel’s transmembrane segments. So, it is unlikely that these nanoparticles interact directly with a relatively small binding pocket for GSK1016790A, unless this would be located on the channel’s outer interface. However, to the best of our knowledge, the binding site for GSK1016790A is not yet known. On the other hand, we gained some insight into this issue from the result that SiNPs also strongly inhibit TRPV4 activation by 4αPDD, a compound that was reported to interact with an internal pocket of the channel formed between transmembrane segments 3 and 4 [44]. Notably, SiNPs also altered the response of TRPV1 to capsaicin, which was reported to bind to an occluded region of this channel [45]. Thus, according to our reasoning above, SiNPs seem not to act on TRPV4 and TRPV1 activation mechanisms by competitive inhibition. At this point we may just speculate that SiNPs induce mechanical perturbations in the plasma membrane that may disrupt activation of TRPV4 and enhance activation of TRPV1.

An interesting observation was that the SiNPs failed to completely inhibit TRPV4 activation. This could result from the presence of two channel populations with distinct sensitivities to SiNPs. This might be the case for the HEK293T cells, in which an endogenous human TRPV4 channel population may co-exist with the transfected mouse TRPV4 channels. However, we observed the lack of complete inhibition also in native 16HBE and mTEC, for which there is no evidence for separate populations of TRPV4. Although further studies are required to address this point, we may also consider that if SiNPs inhibit channel activation by inducing mechanical perturbations in the plasma membrane, these might not be sufficient to completely silence channel activity.

A concomitant finding in our experiments was that SiNPs induce an increase in basal [Ca^2+^]_i_ in the human-derived cells. However, our data demonstrates that TRPV4 channels are not involved in this effect (e.g., lack of inhibitory effect of the TRPV4 blocker HC067047). This is different from what was previously suggested by Gilardino et al., who found that the unspecific TRPV channel blocker ruthenium red inhibited intracellular Ca^2+^ responses to SiNPs [38]. A possible cause for this is that these authors used particles of 50 nm in diameter, which represents about a 170-fold larger volume than that of the ones we used here. Considering that TRPV4 can be activated by mechanical stress at the plasma membrane [39, 46], it is conceivable that only the larger particles may induce TRPV4 activation. On the other hand, Gilardino et al. [38] did find TRPV4-independent responses to SiNPs, which could be triggered via mechanisms similar to those underlying the responses we found in 16HBE cells and in HEK293T cells displaying low basal Ca^2+^ concentration. Of note, for some yet unclear reasons mTEC did not display Ca^2+^ responses upon SiNPs application.

Other features of our results are also qualitatively comparable to those obtained by Gilardino et al. [38]. For instance, the intracellular Ca^2+^ responses to SiNPs occurred after a significant delay and showed a transient initial phase (Fig. 2b). The mechanisms underlying these responses remain fully unknown, but could be related to Ca^2+^ release from intracellular stores. However, the fact that we found SiNPs to increase inward and outward basal currents in HEK293T cells is more consistent with enhanced activities of Ca^2+^-permeable channels in the plasma membrane (e.g., the ubiquitously expressed TRPM7 channels) [47]. These mechanisms should be addressed in future studies because they may bare relevance for the toxic effects of SiNPs (Ca^2+^ overload) in airway epithelial cells.

## Conclusions

Our results show that SiNPs inhibit TRPV4 activation, and that this effect may impair the positive modulatory action of the stimulation of this channel on the ciliary function in airway epithelial cells. It has been proposed that inhibition of TRPV4 could have therapeutic benefits in several respiratory conditions, such as chronic heart failure, hypoxia-induced pulmonary hypertension, acute lung injury, chronic obstructive pulmonary disease and cough [48–51]. However, TRPV4 activity has been shown to underlie protective responses in airway epithelial cells, including the increase in CBF [26, 43]. In addition, TRPV4 function was reported to be important for essential functions in other cells that are direct targets of polluting SiNPs. These include the enhancement of barrier function is skin keratynocytes [52, 53], endothelium-dependent vasorelaxation in pulmonary arteries [54], and ATP release from oesophageal keratynocytes [55]. Thus, inhibition of TRPV4 by SiNPs is expected to have complex effects on airway pathophysiology and rather certain detrimental effects on several epithelial cell functions. Our findings unveil TRPV4 and TRPV1 as defined molecular targets of SiNPs, and prompt for further exploration of the role of these channels in the cellular effects of other types of particulate matter.
